# An exploration of parental age in relation to parents’ psychological health, child adjustment and experiences of being an older parent in families formed through egg donation

**DOI:** 10.1016/j.rbmo.2022.03.029

**Published:** 2022-08

**Authors:** Vasanti Jadva, Joanna Lysons, Susan Imrie, Susan Golombok

**Affiliations:** a Centre for Family Research, University of Cambridge, Free School Lane, Cambridge CB2 3RQ, UK; b Institute for Women's Health, Faculty of Popu​lation Health Sciences, University College London, 86–96 Chenies Mews, London WC1E 6HX, UK

**Keywords:** Advanced parental age, Assisted reproduction, Child adjustment, Egg donation, IVF, Parenting

## Abstract

**Research question:**

Is parental age associated with parents’ psychological health, couple relationship satisfaction and child adjustment in egg donation families, and how do parents think and feel about their age in relation to parenting?

**Design:**

Seventy-two families with a child born after IVF egg donation were included. Mothers were aged between 32 and 51 years and fathers between 31 and 61 years when the target child was born. When the child was aged 5 years, parents were interviewed and asked to complete questionnaire assessments of parenting stress, anxiety, depression, marital quality and child adjustment.

**Results:**

Older parents experienced more parenting stress and poorer couple relationship quality than younger parents. No differences were found for child adjustment. Qualitative content analysis of the interviews revealed themes related to ‘health and mortality’, ‘giving and receiving support’, ‘treatment and age’, ‘positive aspects of older parenting’ and ‘other’.

**Conclusions:**

This exploratory study found that older parents experienced greater parental stress and poorer relationship quality. Some parents had concerns about their older age, specifically in relation to their health and mortality. It would be important to follow up these families as the child grows older to understand the child's thoughts and feelings about having older parents. Furthermore, findings should be replicated in a larger sample of families formed through egg donation, which includes more younger mothers who have used egg donation.

## Introduction

Trends in childbearing age continue to change with average maternal and paternal age at first birth steadily rising in industrialized countries (Braverman, 2017; Martin *et al*., 2019; Australian Institute of Health and Welfare, 2020; ONS, 2021). In the UK in 2019, there were 2,390 births to women aged 45 years and over, compared to 1,619 ten years previously. This trend has been augmented by assisted reproductive technology, which increases the chances of older mothers having a child. According to the Human Fertilisation and Embryology Authority, the average age of a patient using IVF was 35.3 years in 2018 (Human Fertilisation and Embryology, 2020). Clinical improvements have led to better success rates for older patients. In 2018, patients aged 40–42 years had a higher chance of a live birth compared with patients aged under 35 years in 1991, with success rates of 11% and 9% per embryo transferred, respectively (HFEA, 2020). Donor eggs can result in higher birth rates for older patients who can achieve success rates of over 25% using eggs from a younger donor (HFEA, 2020).

The consequences of advanced parental age for obstetric and paediatric outcomes have been well researched (Bouyer *et al*., 2003; Farr *et al*., 2007; Vohr *et al*., 2009; Vaughan *et al*., 2014); however, relatively little is known about the effects of advanced parental age when children are in early to middle childhood. Studies that have examined older parental age in the context of early childhood have focused almost exclusively on its association with child physical and neuropsychiatric outcomes, such as autism spectrum disorder (Glasson *et al*., 2004; Wu *et al*., 2017), schizophrenia (Buizer-Voskamp *et al*., 2011; Byars and Boomsma, 2016) and attention deficit hyperactivity disorder (Chudal *et al*., 2015; D'Onofrio *et al*., 2017; Maitra and Mukhopadhyay, 2019). Moreover, studies on advanced parental age have tended to focus on mothers, although a shift towards the inclusion of fathers in recent years has taken place (Saha *et al*., 2009; Byars and Boomsma, 2018). A Danish cohort study found an increased risk of psychiatric disorders in children of older fathers (>45 years) (McGrath *et al*., 2014). Older paternal age has been found to be associated with reduced fertility, some cancers and schizophrenia (Bray *et al*., 2006). In contrast, studies examining the association between maternal age and child outcomes have found mixed results. An Australian cohort study found that older motherhood was associated with a small increased risk in children at age 5 years in relation to their physical health, wellbeing, social competence and emotional maturity (Falster *et al*., 2018). In a study that examined data from two large UK longitudinal cohorts, however, it was concluded that increasing maternal age was associated with improved health and development for children up to the age of 5 years (Sutcliffe *et al*., 2012). In Denmark, older maternal age was associated with fewer behavioural and socio-emotional problems in children aged 7 and 11 years, although not at age 15 years (Trillingsgaard and Sommer, 2016).

It has been suggested that older parents may experience lower levels of social support, as their friends and family will be relatively older and, therefore, potentially less available or able to provide assistance (Bornstein *et al*., 2006; Mac Dougall *et al*., 2012). Moreover, older parents may find it more difficult to meet the physical demands of parenting young children and may be more preoccupied with their own mortality than their younger counterparts (Dobrzykowski and Stern, 2003; Braverman, 2017; Meyer, 2020). Older parents may also experience stigma around older parenthood (Mac Dougall *et al*., 2012). All these factors may have implications for older parents’ psychological health which, consequently, may adversely affect the child. Qualitative studies have reported some disadvantages associated with older parenthood. In the study by Mac Dougall *et al*. (2012) older mothers (≥ 40 years), reported that parenting in their forties meant they had less energy to dedicate to parenting their young children (mean age 3.5 years). Several studies have also reported older mothers’ perceived lack of social support, particularly from extended family, during infancy (Bornstein *et al*., 2006; Mori *et al*., 2014) and into early childhood (Meyer, 2020). Although older parenthood is becoming increasingly common, significant stigma has been reported around older parenthood, particularly around older motherhood (Campo-Engelstein *et al*., 2016). Media portrayals of older parenthood as ‘selfish’ and ‘dangerous’ may affect older parents’ psychological wellbeing (Campo-Engelstein *et al*., 2015; Jarvie *et al*., 2015). Several qualitative studies have documented older parents’ experiences of stigma from sources such as friends or acquaintances (Friese *et al*., 2006; Jarvie *et al*., 2015) and healthcare professionals (Nottingham-Jones *et al*., 2020; Meyer, 2020). Mac Dougall *et al*. (2012) found that older parents anticipated that their children would receive future stigma for having older parents, although they also reported that having relationships with younger parents had the benefit of keeping them more culturally relevant. A qualitative study of 20 older mothers (mean age at birth 45 years) found that mothers perceived their older age to negatively affect their relationship with their child and their parenting practices (Chen and Landau, 2015). Mothers who represented themselves as more critical, anxious and over-protective within the mother–child relationship perceived these characteristics to be associated with parenting at an older age. Mothers in this study were reported to represent their children as ‘preoccupied’ with their parents’ mortality, which seemed to affect the mother–child relationship.

Conversely, older parents may benefit from greater financial stability and workplace flexibility than their younger counterparts (Mac Dougall *et al*., 2012). Moreover, it has been suggested that older parents may benefit from greater psychological maturity (Camberis *et al*., 2016) and preparedness for parenthood (Proudfoot *et al*., 2009; Mac Dougall *et al*., 2012). Older fathers have been found to be more highly involved in parenting than their younger counterparts (Cooney *et al*., 1993), and older mothers have been found to share more parenting tasks with, and rely more upon, their partners during early infancy (Bornstein *et al*., 2006). One of the difficulties in drawing conclusions from published research on parenting and age is the lack of consensus about what age constitutes ‘older parenthood’, making it difficult to compare results across studies (Boivin *et al*., 2009). With mothers, the threshold for being classed as an older first-time mother ranges from 30 years (Fergusson and Woodward, 1999) to over 40 years (Oldereid *et al*., 2018). Studies also differ in their approach in that some create young/old age groups, whereas others examine age as a continuous variable.

The present study examined families who had used donor eggs in their fertility treatment. The families were part of a larger investigation of heterosexual couples who had conceived a child using IVF with egg donation or own gametes (Imrie *et al*., 2019a). As the treatment of postmenopausal women is often through the use of donor eggs, the study of families who have undergone egg donation lends itself to the examination of the effect of older parenthood, i.e. those who become parents in their late forties or early fifties on parent psychological health, child adjustment and experiences. In the UK there are no legal restrictions on the upper age limit for fertility treatment; clinics can impose their own limits taking into account the risks of treatment. Therefore, focusing on families who have used egg donation provides the opportunity to explore how women of very advanced maternal age, i.e. those aged 45 years or over, experience parenting, and whether they differ from younger parents in relation to psychological health, couple relationship quality and child adjustment.

To our knowledge, only one study has examined the relationship between parental age and child outcomes among children born after assisted reproduction. A survey conducted by Boivin *et al*. (2009) examined the association between maternal age (<31 years, 31–38 years and >38 years), family environment and parent and child wellbeing among families with a child aged 4–11 years (mean 7 years). The study included mothers and fathers who had used assisted conception, including IVF, intracytoplasmic sperm injection and donor insemination, and found that older mothers and fathers reported less warmth towards their partner and more depressive symptoms than younger groups of parents. In terms of child outcomes, the study found no differences in child wellbeing between children of older and younger parents, leading to the conclusion that older parenthood using assisted conception was not associated with negative outcomes for the child (Boivin *et al*., 2009).

It is well documented that infertility treatment can produce psychological strain, which may diminish parents’ psychological resources when they eventually become parents (Redshaw *et al*., 2007). Studies that have compared parenting in families that include children born using IVF with those that include a child born after natural conception have found that mothers who had undergone IVF were possibly less confident in their parental role during the early months of parenthood (McMahon *et al*., 1997). By school age, however, differences between families that had undergone IVF and those that had conceived naturally tended to find more positive parenting among parents who had undergone IVF (Golombok, 2019). These better outcomes may result from IVF parents showing a greater commitment to parenting given their long wait to have a child. Parents who use fertility treatment tend to be of a higher socioeconomic status given that treatment can be costly (Imrie *et al*., 2021). Therefore, mothers and fathers who become parents after assisted reproduction may have different experiences to parents who conceive naturally.

## Materials and methods

### Participants

The original sample of 85 families that had undergone egg donation was recruited through 12 fertility clinics in the UK. Further details on recruitment can be found in Imrie *et al*. (2019a). These families were seen when the target child was aged 1 year, together with a comparison group of 65 families who had conceived using IVF with their own gametes. Findings from the first phase of the study have been reported elsewhere (Imrie *et al*., 2019a; 2019b; 2020). Families were re-visited when the target child was aged 5 years. Data on children's perspectives on their relationships with their parents at age 5 years have been reported elsewhere (Imrie *et al*., 2021). The present paper only reports data from the families who had used egg donation, as parents in families who used their own gametes were significantly younger. Seventy-two families who had used egg donation took part in the second phase of the study, representing a response rate of 85%. Not all fathers were interviewed, with some fathers completing questionnaire assessments only. Demographic information on fathers was collected during the mothers’ interviews.

### Procedure

Trained psychologists visited the families at home. Data were collected through standardized interviews and questionnaires. Mothers and fathers were interviewed separately using a semi-structured interview, which assessed parenting quality and experiences specific to egg donation. Informed written consent was obtained from all participants. Ethical approval was granted by the University of Cambridge Psychology Research Ethics Committee on 22 January 2018 (reference: PRE:2017.097).

### Measures

#### Parent psychological health

Mothers and fathers were asked to complete the short form of the Parenting Stress Index (PSI) (Abidin, 1990), a standardized assessment of stress associated with parenting, with higher scores reflecting greater parenting stress. Test–retest reliability for the total score of the PSI has been reported to be 0.96 over a 1–3-month interval and 0.65 over 1 year. Cronbach's alpha was 0.94 for mothers and 0.93 for fathers. The Trait Anxiety Inventory (TAI) (Spielberger, 1983) was completed by mothers and fathers to measure their general level of anxiety. Internal consistency coefficients have ranged from 0.86 to 0.95 and test–retest reliability coefficients have ranged from 0.65 to 0.75 over 2 months (Spielberger *et al*., 1983). It has been shown to have good reliability and to discriminate well between clinical and non-clinical samples (Spielberger, 1983). Cronbach's alpha was 0.87 for mothers and 0.90 for fathers. Mothers and fathers completed the Edinburgh Depression Scale (Thorpe, 1993) to assess parents’ level of depression, with higher scores indicating greater levels of depression. The Edinburgh Depression Scale has been reported to have satisfactory validity, split-half reliability and is sensitive to changes in depression over time (Cox *et al*., 1987). Cronbach's alpha was 0.87 for mothers and 0.84 for fathers. Mothers and fathers also completed the Golombok Rust Inventory of Marital State (Rust *et al*., 1990), a questionnaire assessment of the quality of the marital relationship, with higher scores indicating poorer marital quality. Split-half reliability was 0.91 for men and 0.87 for women. The Golombok Rust Inventory of Marital State has been shown to discriminate between couples who are about to separate and those who are not (Rust *et al*., 1990). Cronbach's alpha was 0.93 for both mothers and fathers.

#### Child adjustment

The Strengths and Difficulties Questionnaire (SDQ) (Goodman, 1994, 1997) was administered to mothers and fathers to examine child psychological problems. The SDQ produces a total score of the child's adjustment, in which higher scores represent greater difficulties. The SDQ has been shown to have good internal consistency, test–retest reliability and interrater reliability (Goodman, 1994; 1997; 2001; Stone *et al*., 2010). Cronbach's alpha was 0.75 for mothers and 0.81 for fathers.

#### Parents’ thoughts and feelings about age in relation to parenting

Parents were not directly asked any questions specific to their age and parenting. Instead, the interview transcripts were examined for any discussion about age, and any relevant quotations were extracted to a separate excel spreadsheet.

#### Analysis plan

First, scores for mothers and fathers were averaged to obtain mean parental scores for each of the variables of interest in the study. The sample was divided into four groups based on maternal age. Older mothers were defined as those aged 45 years or over when they gave birth to the target child. These mothers were compared with younger groups of mothers stratified by maternal age: those aged 35 years or under, those aged 36–40 years and those aged 41–44 years.

Differences between these four groups were examined using non-parametric Kruskal–Wallis tests. Where significant differences were found, these were followed up with Mann–Whitney U tests to compare each younger age group with the oldest age group aged 45 years and over. Pearson's r correlations were conducted to examine associations between age and the number of IVF cycles couples had undergone and the total amount of time taken to conceive.

Qualitative content analysis (Krippendorf, 2013) of the interview transcripts was conducted to understand participants’ experiences of parenting as older parents. Transcripts were carefully read and any quotes relevant to age in relation to parenting were moved to a separate excel spreadsheet. These data were then coded using an inductive approach to allow new insights to emerge (Hsieh and Shannon, 2005). The codes were collapsed and data-driven categories were produced. These were then checked by a second coder. The main categories are presented below, along with frequency counts and illustrative quotes.

## Results

Most mothers and fathers were first-time parents (58 [80.6%] mothers and 52 [72.2%] fathers). Mothers were aged between 32 and 51 years and fathers between 31 and 61 years when the target child was born. Mothers aged 45 years and above were more likely to have had previous children (Fisher's exact test = 0.042), with eight out of 14 (57.1%) mothers with previous children being aged 45 years or over. An association was also found between fathers’ age and previous children (chi-squared [2, 72] = 7.12; *P* = 0.037), with 10 out of 20 fathers (50%) with a previous child being aged 45 years or over. Mother's age was highly positively correlated with father's age (Pearson's r = 0.56; *P* < 0.001). All parents identified their ethnicity as white. Sample characteristics can be seen in Table 1. The breakdown by age category for the number of IVF cycles couples had undergone to conceive the target child and the amount of time taken in months is presented in Table 2. Neither variable was correlated with mother's age (number of IVF cycles: Pearson's r = 0.08; *P* = 0.51; time taken to conceive: Pearson's r = 0.056; *P* = 0.64).

**TABLE 1:** SAMPLE CHARACTERISTICS OF PARTICIPANTS

Age, years	Mean (range)	SD
Mother	47.10 (38–57)	4.47
Father	48.13 (37–67)	6.57
Child	67.38 (61–82)	4.3
	*n*	%
Mothers age at child's birth, years		
<36	8	11.1
36–40	18	25
41–44	26	36.1
>44	20	27.8
Mother's marital status		
Married	60	83.3
Cohabiting partner	8	11.1
Divorced/separated	3	4.2
Missing	1	1.4
Mother's working status		
Working full-time	22	30.6
Working part-time	36	50
Not currently working	10	13.9
Other	2	2.8
Missing	2	2.8
Father's working status		
Working full-time	57	79.2
Working part-time	10	13.9
Retired	3	4.2
Missing	2	2.8
Financial difficulties		
None	63	87.5
Some	6	8.3
Yes	1	1.4
Missing	2	2.8

**TABLE 2:** EXPERIENCES OF FERTILITY TREATMENT BY MATERNAL AGE

Variables	<36 years	36–40 years	41–44 years	>44 years
Total number of IVF cycles^a^	2.88	3.56	3.73	3.40
Time taken to conceive, months^b^	48.00	66.11	56.05	65.18

Values reported as mean.

a Based on target child.

b From time couple started trying to conceive.

### Parents’ psychological health

Overall, 15 (22.7%) mothers and 15 (24.6%) fathers scored at or above the clinical cut-off for depression, and eight (12.1%) mothers and 10 (16.4%) fathers scored above the cut-off for high anxiety. Twenty-one (34.4%) mothers reported below average relationship satisfaction, including five (8.2%) mothers who reported severe relationship problems and three (4.9%) mothers who reported very severe relationship problems. Six (10%) fathers reported average relationship satisfaction and 12 (20%) fathers reported below average relationship satisfaction.

The median and interquartile range for parents’ psychological health variables is presented in Table 3. Significant differences by age were found for mean parenting stress (*H* [3] = 13.79; *P* = 0.003), mean relationship satisfaction (*H* [3] = 11.66; *P* = 0.009) and mean anxiety (*H* [3] = 7.65; *P* = 0.054) but not for mean depression (*H* [3] = 5.92; *P* = 0.115). Mann–Whitney U tests comparing parents aged 45 years and over with each of the younger age groups found older parents to experience greater parenting stress than those aged 35 years and under (*U* = 24.0; *P* = 0.006) and 41–44 years (*U* = 127.5; *P* = 0.014).

**TABLE 3:** MEDIAN AND INTERQUARTILE RANGE FOR PARENT SCORES ON PSYCHOLOGICAL HEALTH VARIABLES AND CHILD ADJUSTMENT

Psychological assessment scale	Group 1 (35 years and under)			Group 2 (36–40 years)			Group 3 (41–44 years)			Group 4 (45 years and over)		
	Median	IQR	*n*	Median	IQR	*n*	Median	IQR	*n*	Median	IQR	*n*
Parents’ psychological health												
TAI	32.5	7.1	7	38.0	7.6	15	34.5	6.4	21	38.5	13.8	16
EDS	5.5	5.5	7	8.0	5.0	15	4.8	3.5	21	6.8	6	17
PSI/SF	53.8	19.3	7	75.5	24.5	15	64.3	17.4	21	74.5	32.1	16
GRIMS	21.5	28	7	44.6	16.3	14	31.5	24.5	21	41.8	15.5	15
Child adjustment												
SDQ	7.3	6.8	7	9.0	7.3	16	7.0	3.0	20	8.5	7.6	15

EDS, Edinburgh Depression Scale; GRIMS, Golombok Rust Inventory of Marital State; IQR, interquartile range; PSI/SF, Parenting Stress Index; SDQ, Strengths and Difficulties Questionnaire; TAI, Trait Anxiety Inventory.

For relationship satisfaction, parents in the oldest age group experienced poorer relationship satisfaction compared with parents aged 35 years and under (*U* = 26.0; *P* = 0.011) and those aged 41–44 years (*U* = 97.5; *P* = 0.006). For anxiety, none of the younger groups differed significantly from parents aged 45 years and over.

### Child adjustment

First, the SDQ scores for mothers and fathers were looked at separately to examine the number of children who scored above the threshold for probable psychiatric disorder. SDQ score for mothers: two out of 66 (3.0% of the sample) scored above the threshold; SDQ score for fathers: six out of 59 (10.2) scored above the threshold. A Kruskal–Wallis test comparing the mean SDQ for parents in the four age groups was not significant (H [3] = 6.286; *P* = 0.098).

### Parents’ thoughts and feelings about age in relation to parenting

Two-thirds of the available transcripts (48/72 mothers’ interviews and 37/56 fathers’ interviews) were randomly selected for analysis. Of these, 31 (64.6%) mothers and 19 (51.4%) fathers mentioned their age in relation to parenting during the interview. Whether or not age was mentioned during the interview by age of parents is presented in Table 4.

**TABLE 4:** WHETHER OR NOT PARENTAL OLDER AGE WAS MENTIONED DURING THE INTERVIEW BY MATERNAL AGE

Parent	Age mentioned during the interview	Mothers age at birth, years				Total
		<35	36–40	41–44	>45	
		*n*	*n*	*n*	*n*	
Mother	Yes	2	9	13	7	31
	No	4	3	4	6	17
						
Father	Yes	2	5	9	3	19
	No	3	8	4	3	18

Based on transcripts from 48 mothers and 37 fathers.

#### Experiences of older parenting

Parents’ comments about their age in relation to parenting were grouped into five overarching themes: ‘health and mortality’, ‘giving and receiving support’, ‘treatment and age’, ‘positive aspects of older parenting’ and ‘other’ (Table 5).

**TABLE 5:** EXPERIENCE OF MOTHERS AND FATHERS AND FEELINGS ABOUT THEIR AGE IN RELATION TO PARENTING

Feelings	Father (*n* = 19)	%	Mother (*n* = 31)	%
Health and mortality				
Worry about dying/child's future without us	5	26.3	8	25.8
Slower/more tired as a parent	8	42.1	4	12.9
Early months/years being difficult	2	10.5	2	6.5
Experiencing poor health	1	5.2	2	6.5
Bigger adjustment for older parents	1	5.2	2	6.5
Giving and receiving support				
Wanting/seeking out support specific to older parents	3	15.8	5	16.1
Giving advice to others about older parenting	3	15.8	2	6.5
Feeling judged, questioned as older parents	2	10.5	3	9.7
Child having older grandparents	1	5.3	2	6.5
Questioning self as an older parent	1	5.3	2	6.5
Treatment and age				
Not being able to have more children	3	15.8	13	41.9
Wanting children earlier/wish they did it sooner	2	10.5	3	9.7
Benefits of having a younger donor	1	5.3	1	3.2
Time pressure of treatment	0	0	1	3.2
Positive aspects of older parenting				
More committed to parenting	5	26.3	5	16.1
Being stricter/firmer/having better values	2	10.5	1	3.2
Keeps me younger	2	10.5	0	0
Older parenting more acceptable now	1	5.3	2	6.5
Other				
Comparison with parents of different ages	5	26.3	4	12.9
Joked about being an older parent	2	10.5	2	6.5
Became a parent ‘late in the game'	3	15.8	1	3.2
Financially more secure	0	0	1	3.2
Worried about being an older parent	0	0	1	3.2

Some parents mentioned more than one aspect of their experiences of older parenting during the interview.

#### Health and mortality

Around one-quarter of mothers and fathers were concerned about their own mortality and their child's future without them, with eight (25.8%) mothers and five (26.3%) fathers who spoke about their age, having mentioned this.

‘Probably the one thing I worry about with (child) at all is the fact that I know that myself and (partner) are obviously a bit older than the average parent. Compare ourselves to parents at school, some of them are on the older side but there's a lot that probably not a lot older than my older son. So, I think like you know if either of us were to have a sudden bout of ill health or worse, she could be left struggling at an early age’.

Father aged 48 years at child's birth

‘Yeah, it's the fact that we're older so you know, we're not going to be here for ever and ever, and um, so yeah how, how is she going to cope with those and, um, yeah will she navigate that?’

Mother aged 50 years at child's birth

Only two (6.5%) mothers and one (5.3%) father were experiencing health problems that they felt were affecting their parenting. Parents also spoke about feeling tired and slow as older parents, with four (12.9%) mothers and eight (42.1%) fathers commenting on this.

‘…it's been quite a benefit being an older parent, but at the same time it's been a bit of a burden being an older parent because we are getting to that stage of we have worked over 20 odd years and we are not as bouncy and as elastic as we might of once been in our lives...’

Mother aged 33 years at child's birth

#### Giving and receiving support

During the interview, some parents (five [16.1%] mothers and three [15.8%] fathers) mentioned wanting help specific to older parenting or to receive specific support. Other parents (two [6.5%] mothers and three [15.8%] fathers) spoke about giving advice to others:

‘We've occasionally, where we've had other, typically women in their late forties coming to us saying oh you are so lucky that you've got a child and all that sort of thing and we explain that it's IVF and they've never even thought of it, and whether or not they'll go and do that process or not I don't know, but you know, the idea that it might be available to somebody who wants a child is something that we wouldn't [want] to deny that we've not took part in it.’

Father aged 53 years at child's birth

A smaller number of parents spoke about their children having older grandparents because of their own older age. A few parents mentioned feeling judged for being an older parent or questioned their own parenting skills because of their age:

‘… not long ago with a bunch of young…well young mums…who were discussing you're not a real mum if you have IVF or be helped, and you're definitely not a real mum if you have a caesarean and don't breast feed. Well, you know, I've had IVF and a caesarean and I didn't breast feed, I mean... I just left, haha.’

Mother aged 43 years at child's birth

#### Treatment and age

Far more mothers than fathers (13 [41.9%] mothers and three [15.8%] fathers) spoke about not being able to have more children because of their age. For some, these discussions became quite emotional:

‘Um, and, I just wish we'd been given the opportunity earlier (for testing) (crying) because there could have been more than one, and there is, we've got a frozen embryo but because of our ages and I was quite poorly after I'd had [child], not mentally, physically, because I had lost quite a lot of blood, we made the decision not to put it back.’

Mother aged 41 years at child's birth

A few parents, like the mother in the quote above, also mentioned wishing they had become parents sooner. One mother and one father spoke about the benefits of having a younger egg donor. This included a perception that there would be fewer risks associated with the pregnancy and that the donor could take the place of a parent in the future.

#### Positive aspects of older parenting

Several parents spoke about the positive aspects of older parenting; these mainly included the greater commitment they could make to parenting as they felt they had passed the phase in their life where they were socialising or going out. For example*,*

‘Yeah because we do notice that other parents want to have their own sort of life as it was before they had children, or they want to go out lots and stuff, party or whatever, I don't know whether it's because we're older or whether it's because we struggled for so many years to have him, but we think everything we do with him makes it a better experience and…we're doing things for him and that‘s given us a reason for going to these places and seeing these things and…and to see his face light up and…you know, that just enhances everything in life to enjoy it all together as a family.’

Mother aged 39 years at child's birth

Parents also said that being a parent kept them young and felt that older parenting is more acceptable now than previously.

#### Other

Some parents mentioned other thoughts on being an older parent, including comparing themselves with parents of different ages, without seeing this as an advantage or disadvantage, using humour to describe their experiences of being an older parent and feeling like they have come to parenting ‘late in the game’ (Table 5).

## Discussion

Findings from this study should be interpreted with caution because of the small sample sizes of the subgroups of parents who fell into the different age groups. Nevertheless, to our knowledge, this is the only sample in which families with mothers of an advanced age of over 45 years have been compared with younger mothers who have used the same method of conception. Egg donation is a form of assisted conception that enables older women to achieve motherhood, and thus studying the experiences of families who have used egg donation can provide useful insights into the outcomes of mothers of an advanced age. Given that women are far less likely to conceive without the help of medical intervention at this age, the present study does not claim to be representative of older mothers in the general population.

At age 5 years, the children of older parents did not experience psychological problems as rated by parents. The individual scores from mothers and fathers also showed that most children in the sample were not experiencing adjustment problems. This finding is consistent with that found by Boivin *et al*. (2009) in a sample of children born after IVF, intracytoplasmic sperm injection and donor insemination. The present sample includes an older group of parents in whom the mother was aged 45 years and over. Therefore, the findings from the present study extend what is known already by showing that children of an older group of parents do not seem to experience social or emotional difficulties at the age of 5 years.

Relationship quality was found to differ by parental age, with poorer couple relationships among older parents. This finding is similar to that observed by Boivin *et al*. (2009), and likely reflects the decline in relationship quality over time that has been found among marriages in general (Van Laningham *et al*., 2001). For example, Umberson *et al*. (2005) followed up couples over an 8-year period and found that, although older people had higher levels of marital quality at the start of their study, all age groups showed a decline in marital quality over time. Therefore, the finding that older parents experience poorer relationship satisfaction is not surprising and is consistent with the wider research on changes to couple relationships over-time.

To the best of our knowledge, this was the first study to include mothers of an advanced maternal age of 45 years and over. Indeed, some of the mothers and fathers in this study had had their child in their early fifties. Older parents were found to experience greater parenting stress than their younger counterparts. Studies have found that older parents may find it more difficult to meet the physical demands of parenting young children and may be more preoccupied with their own mortality than their younger counterparts (Dobrzykowski and Stern, 2003; Braverman, 2017; Meyer, 2020). The qualitative data showed that some parents wished for greater support and some experienced feelings of being judged as older parents, and feelings of self-doubt. Furthermore, some parents had concerns about their own health and mortality. Therefore, these additional factors may explain why older parents experience greater parenting stress compared with younger parents. The findings point to a need for additional support tailored for older parents.

Egg donation carries a number of medical risks, including an increased risk of hypertensive disorders of pregnancy, pre-eclampsia, low birth weight, preterm births and a greater likelihood of obstetric emergencies (Berntsen *et al*., 2021). The present study found that these risks may not be understood by recipients themselves. For example, one mother felt there would be fewer health risks for the baby given the donor was younger than the mother. These factors should be explored in future studies looking at older parenting in relation to gamete donation specifically. More broadly, patients’ understanding of the risks associated with egg donation is an underexplored area of research.

As mentioned earlier, the outcomes and experiences of families who have undergone assisted reproductive technology may not be generalizable to the general population. First, treatment is expensive and patients undergoing IVF tend to come from a higher socioeconomic status than families in the general population. Indeed, in the present study, only one family reported experiencing financial difficulties. Furthermore, given that it is up to clinics to use their discretion about whether a couple can receive treatment, it is possible that some form of selection is taking place, whereby healthier men and women may be more likely to be treated. Moreover, healthier patients may be those whose treatments are more likely to be successful. Although several parents were concerned about their health and mortality, only a few said that their age was related to poor health at the time of the study.

It is also important to note that the participants in this study were on average much older than parents within the general population. Given that egg donation is itself a treatment more commonly used by older women, the present study had few women in their early thirties, and none aged in their twenties. It is possible, that more differences may be found when comparing women of a very advanced maternal age with those of a much younger age. Therefore, the need for a larger sample of families formed through egg donation with a broader age range of mothers should be an area of future focus.

The present study did not ask parents directly about their older age; this can be considered both an advantage and limitation of the study. On the one hand, by not asking all parents, it is not clear if other parents had any concerns that they did not have the opportunity to raise. On the other hand, by not asking the question directly, parents who felt that age was an issue in terms of their parenting may have brought this into the conversation when relevant. Therefore, these data may be more ecologically valid. Of interest is that parents aged in their early thirties also talked about their older age, reinforcing the idea that older parenting is a relative concept that holds different meaning for individuals. This may be influenced by the age of other parents within their direct social sphere. Several parents commented on comparing their age with other parents at the school gates, for example. Nonetheless, future studies discussing parental age with parents of all ages would provide new insights into the experiences of age and parenting.

In conclusion, it is important to remember that children in the study were still young, and the parents were also relatively young. Older parenting raises concerns about the additional burdens and stresses a child could experience in caring for elderly parents and those with poor health. The mean age of mothers and fathers of 5-year-olds in the present study was 47 and 48 years respectively, with the oldest parents aged 57 and 67 years. Older mothers who on average had their child at the age of 45 years have reported their children (aged on average 8.5 years (range 3–17) to be preoccupied by their mortality (Chen and Landau, 2015); given that parental mortality was a concern for parents in the present study, it is important to understand the child's thoughts and feelings about this. Following up these children as they grow older is important to better understand the effect of older parental age on child outcomes and experiences throughout childhood and beyond.

## References

[bib0001] Abidin R. (1990).

[bib0003] Australian Institute of Health and Welfare (2020). Australia's mothers and babies 2018: in brief. Perinatal Statistics Series.

[bib0004] Boivin J., Rice F., Hay D., Harold G., Lewis A., van den Bree M.M., Thapar A. (2009). Associations between maternal older age, family environment and parent and child wellbeing in families using assisted reproductive techniques to conceive. *Social science & medicine* (1982).

[bib0005] Bornstein M.H., Putnick D.L., Suwalsky J.T.D., Gini M. (2006). Maternal Chronological Age, Prenatal and Perinatal History, Social Support, and Parenting of Infants. Child Development.

[bib0006] Bouyer J., Coste J., Shojaei T., Pouly J.L., Fernandez H., Gerbaud L., Job-Spira N. (2003). Risk factors for ectopic pregnancy: A comprehensive analysis based on a large case-control, population-based study in France. American Journal of Epidemiology.

[bib0007] Berntsen S., Larsen E.C., la Cour Freiesleben N., Pinborg A. (2021). Pregnancy outcomes following oocyte donation. Best practice & research. Clinical obstetrics & gynaecology.

[bib0008] Braverman A.M. (2017). Old, older and too old: age limits for medically assisted fatherhood?. Fertility and Sterility.

[bib0009] Bray I., Gunnell D., Davey Smith G (2006). Advanced paternal age: how old is too old*?*. Journal of epidemiology and community health.

[bib0010] Byars S.G., Boomsma J.J. (2016). Opposite differential risks for autism and schizophrenia based on maternal age, paternal age, and parental age differences. Evolution, Medicine and Public Health, 2016.

[bib0011] Camberis A.-L., McMahon C.A., Gibson F.L., Boivin J. (2016). Maternal Age, Psychological Maturity, Parenting Cognitions, and Mother–Infant Interaction. Infancy.

[bib0012] Campo-Engelstein L., Santacrose L.B., Master Z., Parker W.M. (2016). Bad moms, blameless dads: The portrayal of maternal and paternal age and preconception harm in U.S. newspapers. AJOB Empirical Bioethics.

[bib0013] Chen W., Landau R. (2015). First childbirth and motherhood at post natural fertile age: a persistent and intergenerational experience of personal and social anomaly?. Social work in health care.

[bib0014] Cooney T.M., Pedersen F.A., Indelicato S., Palkovitz R. (1993). Timing of Fatherhood: Is “On-Time” Optimal?. Journal of Marriage and Family.

[bib0017] Dobrzykowski T.M., Stern P.N. (2003). Out of sync: A generation of first-time mothers over 30. Health Care for Women International.

[bib0018] Falster K., Hanly M., Banks E., Lynch J., Chambers G., Brownell M., Jorm L. (2018). Maternal age and offspring developmental vulnerability at age five: A population-based cohort study of Australian children. PLoS Medicine.

[bib0019] Farr S.L., Schieve L.A., Jamieson D.J. (2007). Pregnancy loss among pregnancies conceived through assisted reproductive technology, United States, 1999-2002. American Journal of Epidemiology.

[bib0020] Fergusson D., Woodward L. (1999). Maternal Age and Educational and Psychosocial Outcomes in Early Adulthood. The Journal of Child Psychology and Psychiatry and Allied Disciplines.

[bib0021] Friese C., Becker G., Nachtigall R.D. (2006). Rethinking the biological clock: Eleventh-hour moms, miracle moms and meanings of age-related infertility. Social Science and Medicine.

[bib0023] Golombok S., Bornstein M.H. (2019). Handbook of parenting: Being and becoming a parent.

[bib0024] Goodman R. (1994). A modified version of the Rutter parent questionnaire including extra items on children's strengths: A research note. Journal of Child Psychology and Psychiatry.

[bib0025] Goodman R. (1997). The Strengths and Difficulties Questionnaire: A research note. Journal of Child Psychology and Psychiatry.

[bib0026] Goodman R. (2001). Psychometric properties of the strengths and difficulties questionnaire. Journal of the American Academy of Child and Adolescent Psychiatry.

[bib0028] Hsieh H.F., Shannon S.E. (2005). Three approaches to qualitative content analysis. Qualitative health research.

[bib0030] Imrie S., Jadva V., Fishel S., Golombok S. (2019). Families Created by Egg Donation: Parent–Child Relationship Quality in Infancy. Child Development.

[bib0031] Imrie S., Jadva V., Golombok S. (2019). Psychological well-being of identity-release egg donation parents with infants. Human Reproduction.

[bib0032] Imrie S., Jadva V., Golombok S. (2020). Making the child mine": Mothers' thoughts and feelings about the mother-infant relationship in egg donation families. Journal of family psychology: JFP: journal of the Division of Family Psychology of the American Psychological Association (Division 43).

[bib0033] Imrie R., Ghosh S., Narvekar N., Vigneswaran K., Wang Y., Savvas M. (2021). Socioeconomic status and fertility treatment outcomes in high-income countries: a review of the current literature. Human fertility (Cambridge, England), 1–11. Advance online publication.

[bib0034] Jarvie R., Letherby G., Stenhouse E. (2015). Renewed”“older” motherhood/mothering: A qualitative exploration. Journal of women & aging.

[bib0035] Krippendorf K. (2013).

[bib0036] Mac Dougall K., Beyene Y., Nachtigall R.D. (2012). Inconvenient biology:” advantages and disadvantages of first-time parenting after age 40 using in vitro fertilization. Human Reproduction.

[bib0037] Martin J.A., Hamilton B.E., Osterman M.J.K., Driscoll A.K. (2019). National Vital Statistics Reports. Births: Final Data for 2019.

[bib0038] McGrath J.J., Petersen L., Agerbo E., Mors O., Mortensen P.B., Pedersen C.B. (2014). A comprehensive assessment of parental age and psychiatric disorders. JAMA psychiatry.

[bib0039] McMahon C.A., Ungerer J.A., Tennant C., Saunders D. (1997). Psychosocial adjustment and the quality of the mother-child relationship at four months postpartum after conception by in vitro fertilization. Fertility and sterility.

[bib0042] Meyer D.F. (2020). Psychosocial needs of first-time mothers over 40. Journal of Women and Aging.

[bib0043] Mori E., Iwata H., Sakajo A., Maehara K., Ozawa H., Maekawa T., Saeki A. (2014). Postpartum experiences of older Japanese primiparas during the first month after childbirth. International Journal of Nursing Practice.

[bib0044] Nottingham-Jones J., Simmonds J.G., Snell T.L. (2020). First-time mothers’ experiences of preparing for childbirth at advanced maternal age. Midwifery.

[bib0045] Oldereid N.B., Wennerholm U.B., Pinborg A., Loft A., Laivuori H., Petzold M., Romundstad L.B., Söderström-Anttila V., Bergh C. (2018). The effect of paternal factors on perinatal and paediatric outcomes: a systematic review and meta-analysis. Human reproduction update.

[bib0047] Proudfoot S., Wellings K., Glasier A. (2009). Analysis why nulliparous women over age 33 wish to use contraception. Contraception.

[bib0048] Redshaw M., Hockley C., Davidson L.L. (2007). A qualitative study of the experience of treatment for infertility among women who successfully became pregnant. *Human reproduction* (Oxford, England).

[bib0049] Rust J, Bennun I, Golombok S. (1990). The GRIMS: A psychometric instrument for the assessment of marital discord. Journal of Family Therapy.

[bib0050] Saha S., Barnett A.G., Buka S.L., McGrath J.J. (2009). Maternal age and paternal age are associated with distinct childhood behavioural outcomes in a general population birth cohort. Schizophrenia Research.

[bib0051] Spielberger C. (1983).

[bib0052] Sutcliffe A.G., Barnes J., Belsky J., Gardiner J., Melhuish E. (2012). The health and development of children born to older mothers in the United Kingdom: Observational study using longitudinal cohort data. BMJ (Online).

[bib0053] Stone L.L., Otten R., Engels R.C., Vermulst A.A., Janssens J.M. (2010). Psychometric properties of the parent and teacher versions of the strengths and difficulties questionnaire for 4- to 12-year-olds: a review. Clinical child and family psychology review.

[bib0054] Thorpe K. (1993). A study of the use of the Edinburgh Postnatal Depression Scale with parent groups outside the postpartum period. Journal of Reproductive and Infant Psychology.

[bib0055] Trillingsgaard T., Sommer D. (2016). Associations between older maternal age, use of sanctions, and children's socio-emotional development through 7,11, and 15 years. European Journal of Developmental Psychology.

[bib0056] Umberson D., Williams K., Powers D.A., Chen M.D., Campbell A.M. (2005). As Good as it Gets? A Life Course Perspective on Marital Quality. Social forces; a scientific medium of social study and interpretation.

[bib0057] VanLaningham J., Johnson D.R., Amato P. (2001). Marital happiness, marital duration, and the U-shaped curve: Evidence from a five-wave panel study. Social Forces.

[bib0058] Vaughan D.A., Cleary B.J., Murphy D.J. (2014). Delivery outcomes for nulliparous women at the extremes of maternal age - A cohort study. BJOG.

[bib0059] Vohr B.R., Tyson J.E., Wright L.L., Perritt R.L., Li L., Poole W.K. (2009). Maternal Age, Multiple Birth, and Extremely Low Birth Weight Infants. Journal of Pediatrics.

